# A new species of *Psammonitocrella* Huys, 2009 (Copepoda, Harpacticoida, Ameiridae) from California (USA), with a discussion of the relationship between *Psammonitocrella* and Parastenocarididae

**DOI:** 10.3897/zookeys.996.55034

**Published:** 2020-11-24

**Authors:** Paulo Henrique Costa Corgosinho, Terue Cristina Kihara, Pedro Martínez Arbizu

**Affiliations:** 1 Department of General Biology, Universidade Estadual de Montes Claros, 39401-089, Montes Claros, Brazil Universidade Estadual de Montes Claros Montes Claros Brazil; 2 Senckenberg am Meer Wilhelmshaven, Abt. Deutsches Zentrum für Marine Biodiversität, DZMB, German Centre for Marine Biodiversity Research, Südstrand 44, 26382, Wilhelmshaven, Germany Senckenberg am Meer Wilhelmshaven Wilhelmshaven Germany

**Keywords:** fresh water, groundwater diversity, meiofauna, phylogeny, *Psammonitocrella kumeyaayi* sp. nov., San Clemente Canyon, systematics

## Abstract

The freshwater harpacticoid *Psammonitocrella kumeyaayi***sp. nov.** from the Nearctic Region (California; USA) is proposed. The position of the genus within Harpacticoida and its relationship with the Parastenocarididae is discussed. The new species can be included within *Psammonitocrella* on account of a) the cylindrical furca, longer than the telson, b) the unmodified inner spine on the basis of the male first leg, c) loss of the outer spine on the second exopodal segment of the first leg, d) loss of the outer spine of the third exopodal segment of the second, third, and fourth legs, e) loss of the inner apical seta on the third exopodal segment of the second and third legs, f) transformation of the inner apical seta of the third exopodal segment of the fourth leg into a spine, and g) loss of the endopodite of the fourth leg. The new species differs remarkably from *P. boultoni*, and *P. longifurcata* in the loss of the outer spine of the second exopodal segment of the fourth leg, in the presence of a one-segmented fifth leg exopodite, and in the presence of an outer seta on the basis of the first and second legs. Both *Psammonitocrella* and the known species of Parastenocarididae have a one-segmented endopod on the fourth leg, and the endopods of the second and third legs are reduced to one or two segments. *Psammonitocrella* is currently allocated into the Ameiridae, and evidence suggesting a sister-group relationship with Parastenocarididae—both share the loss of the inner seta on the first endopodal segment of the first leg—indicates that the Parastenocarididae should be included into the Ameiridae. In an evolutionary context, Parastenocarididae could have evolved from a lineage of freshwater ameirids that became interstitial in continental waters and colonized aquifers and groundwaters.

## Introduction

The family Ameiridae Boeck, 1865 is composed of about 300 species accommodated in 47 marine and freshwater genera (Walter and Boxshall 2020), excluding *Anoplosomella* Strand, 1929 and *Malacopsyllus* Sars, 1911, transferred to Argestidae Por, 1986 by Corgosinho and Martínez Arbizu (2010). Nowadays, with 150 freshwater species, this is one of the most species-rich families in fresh water. It is especially diverse in subterranean waters (Rouch 1986; Galassi 2001), and is dominated by the group of genera related to *Nitokra* and *Nitocrella* (Boxshall and Jaume 2000; Boxshall and Defaye 2008). The freshwater species of Ameiridae are found worldwide, with the highest diversity recorded from the Palearctic Region (Boxshall and Defaye 2008).

*Psammonitocrella* Huys, 2009 is known only from the USA, and is represented by *P. boultoni* Rouch, 1992 and *P. longifurcata* Rouch, 1992. The taxonomic position of this genus was debated in the last decades. In their analysis of a wide range of ameirid-like taxa, Martínez Arbizu and Moura (1994) revealed the sister-group relationship between *Psammonitocrella* and Parastenocarididae Chappuis, 1940, and concluded, that, if the family rank of Parastenocarididae is to be maintained, *Psammonitocrella* could not be maintained within Ameiridae. Lee and Huys (2002) proposed to return *Psammonitocrella* to Ameiridae, and Karanovic and Hancock (2009) considered the genus as a derived group within a clade of freshwater ameirids—with one-segmented, reduced or without a P4enp—closely related to *Eduardonitocrella mexicana* (Suárez-Morales & Iliffe, 2005) and *Stygonitocrella orghidani* (Petkovski, 1973) *incertae sedis* (Karanovic and Hancock 2009). None of these authors discussed the position of Parastenocarididae, but by excluding this family from their analyses, and including *Psammonitocrella* into Ameiridae, they implicitly disagreed with the sister-group relationship between Parastenocarididae and *Psammonitocrella*.

In this work we describe *Psammonitocrella kumeyaayi* sp. nov from the historical collection of late Professor W. Noodt, collected by himself in the state of California, USA, in 1974. An amended diagnosis is offered for the genus, and the position of the new species within the genus *Psammonitocrella* is briefly addressed. Here we discuss an alternative hypothesis for the position of Parastenocarididae within Harpacticoida.

## Material and methods

A single male specimen of the new species was sorted from a sample collected by Prof. Wolfram Noodt on 29/03/1974 at a locality identified as San Clemente Canyon (California, USA). The sampling locality is described in Noodt’s field notebook and in the sample identification as “wenig fließendes stehendes Wasser”, an almost lotic or a standing water environment or with very low current. After an extensive toponymic search we concluded that Prof. Noodt was referring to the San Clemente Canyon in San Diego County. The San Clemente Canyon is nowadays included in the Marian Bear Memorial Park in the city of San Diego, a linear open space park along a canyon rich in temporary water bodies.

The habitus was drawn from the whole specimen temporarily mounted onto one slide with glycerin as mounting medium; adhesive plastic discs were used to support the cover slip and prevent destruction of the specimen (Kihara and Rocha 2009). Total length of the only specimen available was measured from the tip of the rostrum to the posterior rim of the furca. Once the habitus was drawn, the specimen was dissected under a Leica MZ12.5 microscope (Leica, Wetzlar, Germany). The dissected parts were mounted on slides using glycerin as mounting medium, and preparations were sealed with transparent nail varnish. Drawings were made at 400× and 1000× magnification with a Leica DM 2500 microscope (Leica, Wetzlar, Germany) equipped with Nomarsky interference contrast and a drawing tube.

The terms ‘furca’ and ‘telson’ are used according to Schminke (1976). Terminology and homologization of maxillary and maxillipedal structure follow Ferrari and Ivanenko (2008). Therefore, by the application of serial homology, the nomenclature of Huys and Boxshall (1991) for the maxilla (fig. 1.5.5, p. 26) is modified as follows: the praecoxa of the maxilla is hereafter recognized as the syncoxa (praecoxa and coxa fused), the coxa is considered as the basis, and the basis is recognized as the first endopodal segment with claw. Other morphological terms follow Huys and Boxshall (1991).

The diagnosis represents the reconstructed ground pattern of *Psammonitocrella*. It is amended from Karanovic and Hancock (2009: 46). The term “ground pattern” is used in the sense of “Grundmuster” (Ax 1984: 156), and refers to all plesiomorphies and autapomorphies present in the stem species of the genus.

Abbreviations used in the text and figures: A1= antennule, A2 = antenna, aes = aesthetasc, ap= apomorphy, benp(s)= basendopod(s), cph = cephalothorax, DAS= distal apical seta; DOS= distal outer seta; enp= endopod, exp(s)= exopod(s), enp1–3 = endopodal segments 1–3, exp1–3 = exopodal segments 1–3, Fu= furca, GF = genital field, IAS= inner apical seta/spine; ms= modified spine, md = mandible, mx1 = maxillule, mx2 = maxilla, mxp = maxilliped, P1–P6= legs 1 to 6, pl= plesiomorphy, Ur1 to 5= first to fifth urosomites.

## Results

### Order Harpacticoida Sars, 1903

**Family Ameiridae Boeck, 1865**

#### Genus Psammonitocrella

Taxon classificationAnimaliaHarpacticoidaAmeiridae

Huys, 2009

C3C6B473-52A0-5F1D-87A0-6F82AFA7F982

##### Diagnosis amended.

Ameiridae. Body small, slender, and cylindrical, without distinct demarcation between prosome and urosome. Integument weakly chitinized, with or without lateral cuticular windows on P2–P3-bearing somites (presence of these cuticular windows is uncertain for *P. longifurcata* and *P. boultoni*); hyaline posterior fringe of all somites smooth. First pedigerous somite incorporated into cephalosome. Prosome ornamented only with sensilla; Ur ornamented with rows of small spinules. Genital (Ur2) and Ur3 separated in female; GF with single large copulatory pore, wide copulatory duct, and two small semicircular seminal receptacles; single small genital aperture covered by fused reduced P6, without armature or ornamentation. Telson unornamented or ornamented with small spinules and tube pores. Anal operculum unornamented or ornamented with small spinules, wide and convex, not reaching or reaching posterior end of anal somite. Fu slender, tapering distally or cylindrical, slightly divergent, longer than anal somite, with long tube pores in *P. kumeyaayi* sp. nov.; seta VII inserted subdistally, close to inner margin, less than half the ramus length; outer setae I and II inserted on the proximal half of Fu; seta III on the same plane as seta VII; seta VI minute; seta V without breaking plane; seta IV longer, as long as or shorter than ramus. A1 long and slender, eight-segmented in female, 10-segmented, haplocer, and geniculate in male; without seta on short first segment in female, with a seta on the first segment in male. A2 composed of coxa, basis, two-segmented enp and one-segmented exp; exp armed with three setae. Md with narrow cutting edge and two-segmented uniramous palp; basis unarmed; enp with three to five apical setae. Mx1 with praecoxal arthrite armed with three distal claws, one or two minute oral setae, and one or two accessory aboral setae; coxa with two or three apical setae; basis with two to four apical setae; enp present or absent. Mx2 with syncoxal endite armed with a single element, or endite absent; basal endite armed with three elements; enp1 drawn out into a claw, with an accessory seta; remaining endopodal segment represented by one or two setae. Basis of P1 with unmodified inner spine in male, and with or without outer seta; without any other sexual dimorphism in swimming legs; basis of P2 with or without outer seta. Enp of P1 three-segmented; enp of P2 and P3 one- or two-segmented, of P4 reduced to small knob or completely absent; P1enp1 unarmed, long, reaching distal margin of exp2 or nearly as long as exp1; P1enp2 unarmed, longer than enp3; P1enp3 with outer seta, with or without geniculation, inner seta probably geniculated in all species; if enp of P2 and P3 two-segmented, then first segment unarmed, second segment with one apical seta. All swimming legs with three-segmented exps; exopodal segments of P2–P4 subequal in length; P1–P4exp1 without inner seta; exp2 of P1 without outer spine and with inner seta; exp2 of P2 and P3 with inner seta and outer spine; exp2 of P4 with or without outer spine and with inner seta; exp3 of P1 with two outer spines, two geniculate distal setae, and without inner armature; P2–P4exp3 without outer spine; P2–P3exp3 with or without inner apical seta; inner apical seta of P4exp3 may be transformed into a spine. P5 similar in both sexes; fused to somite or free; with or without recognizable endopodal lobe, and with recognizable exopodal lobe, or exp one-segmented; endopodal lobe (if present) armed with one or two elements; exopodal lobe or exp with four, three, two or only one seta.

##### Type species.

*Psammonitocrella boultoni* Rouch, 1992.

##### Other species.

*Psammonitocrella longifurcata* Rouch, 1992; *P. kumeyaayi* sp. nov.

**Table 1.** Setal formulae of the swimming legs as hypothesized to occur in the ground pattern of the genus. Roman numerals represent spines; Arabic numerals represent setae.

Legs	Basis	Exopod	Endopod
P1	1-I	I-0, 0-1, II-2-0	0-0, 0-0, 0-I+1^*^-0
P2	1-0	I-0, I-1, 0-I+2^**^-0	0-0, 0-1-0^+^
P3	1-0	I-0, I-1, 0-I+1-0	0-0, 0-1-0^+^
P4	1-0	I-0, I-1^++^, 0-I+1-I^#^	Knob^##^

*2 setae in *P. kumeyaayi* sp. nov; distal outer seta geniculate. ^**^I+1 in *P. boultoni* and *P. kumeyaayi* sp. nov. ^+^one-segmented in *P. longifurcata*; 0-1-0. ^++^0-1 in *P. kumeyaayi* sp. nov. ^#^0-I+1-0 in *P. longifurcata*. ^##^Absent in *P. kumeyaayi* sp. nov. and in *P. longifurcata*.

#### Psammonitocrella kumeyaayi sp. nov.

Taxon classificationAnimaliaHarpacticoidaAmeiridae

2EA1B414-643A-586D-A2CA-3B8A57537D8E

http://zoobank.org/16622CED-8D1F-4F39-9132-E7C66AD3A972

Figs 1 , 2 , 3 , 4

##### Material examined.

***Holotype***: One male dissected and mounted onto 7 slides (reg. no. SMF 37256/1-7; 1-7 refers to the number of slides).

***Type locality***: San Clemente Canyon, San Diego, California, USA (32.8446°N, 117.1949°W).

##### Description of male.

**Figure 1.**
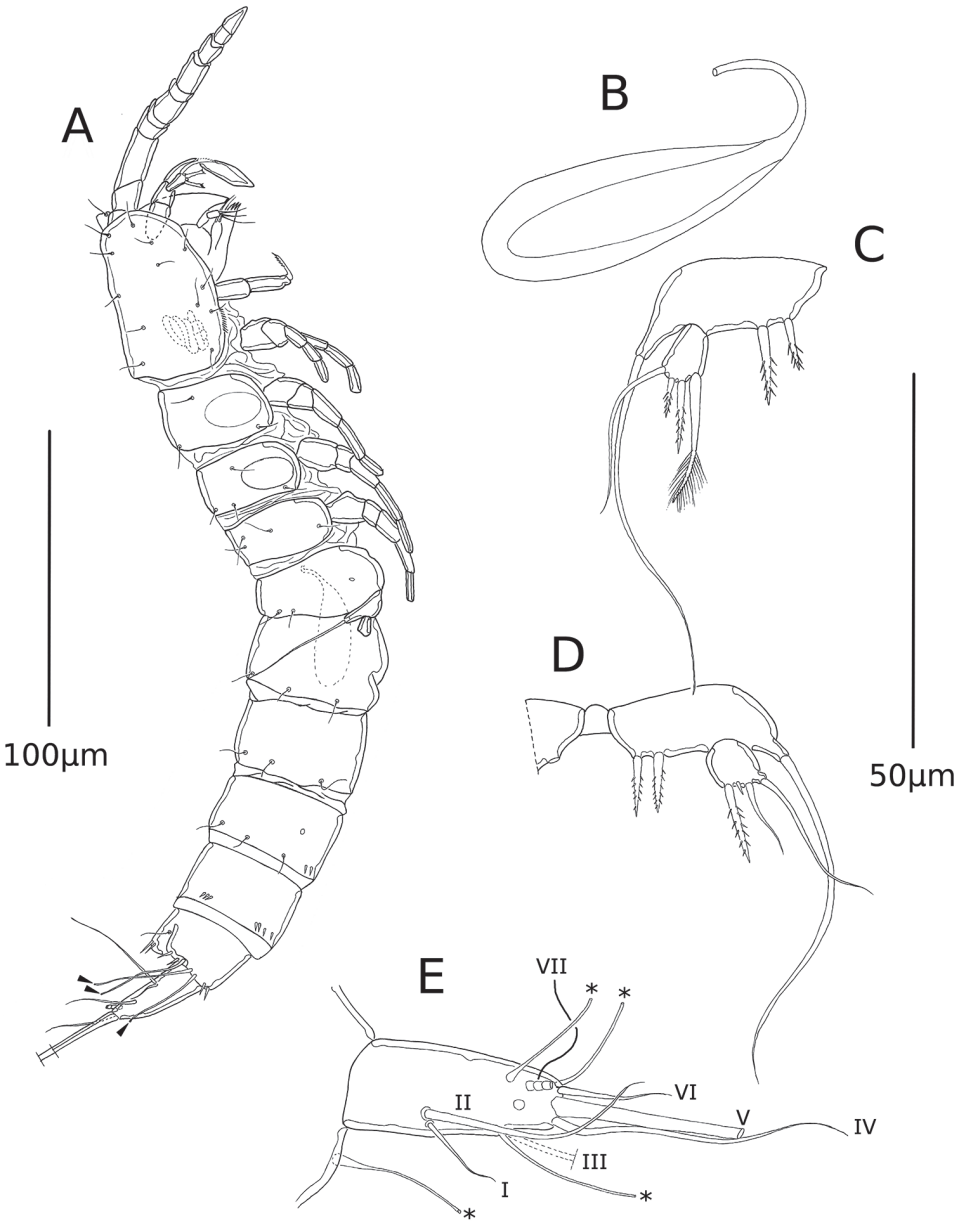
*Psammonitocrella kumeyaayi* sp. nov., male. **A** Lateral habitus of male **B** spermatophore **C** right P5**D** left P5**E** furca. Arrowheads on (**A**) and asterisks on (**E**) mark tube pores. Roman numerals indicate each seta on (**E**).

Total length 302 µm, measured from rostrum to end of furca. Rostrum not fused to cph, with two sensilla on tip (Fig. 1A). First and second free prosomal segments with large oval window on each side of the body (Fig. 1A). Ur4 and 5 weakly ornamented with small spinules. Pattern of sensilla and pores as depicted (Fig. 1A). Telson weakly ornamented with small spinules on anal operculum; laterally with few spinules and three long tube pores (Fig. 1A; tube pores marked with arrowheads). Fu (Fig. 1A, E) cylindrical, tapering distally, approximately 2.5 times longer than wide, 1.4 times longer than telson, with dorsal pore subdistally and three long tube pores –two on outer margin, one on inner margin (marked with asterisk on Fig. 1E) armed with seven setae; setae I and II inserted dorsally on proximal half, seta I shorter; seta III (Fig. 1A, broken in Fig. 1E) on distal third, longer than seta I, shorter than seta II; seta IV inserted distally on outer margin, longer than seta II; seta V longest (broken in Fig. 1A and E); seta VI inserted distally on inner margin, almost as long as seta I; seta VII broken (Fig. 1A and E), tetra-articulated at basis, inserted dorsally at the same level as seta III. Spermatophore (Fig. 1A, B) occupying almost the whole length of Ur4 and 5 combined.

**Figure 2.**
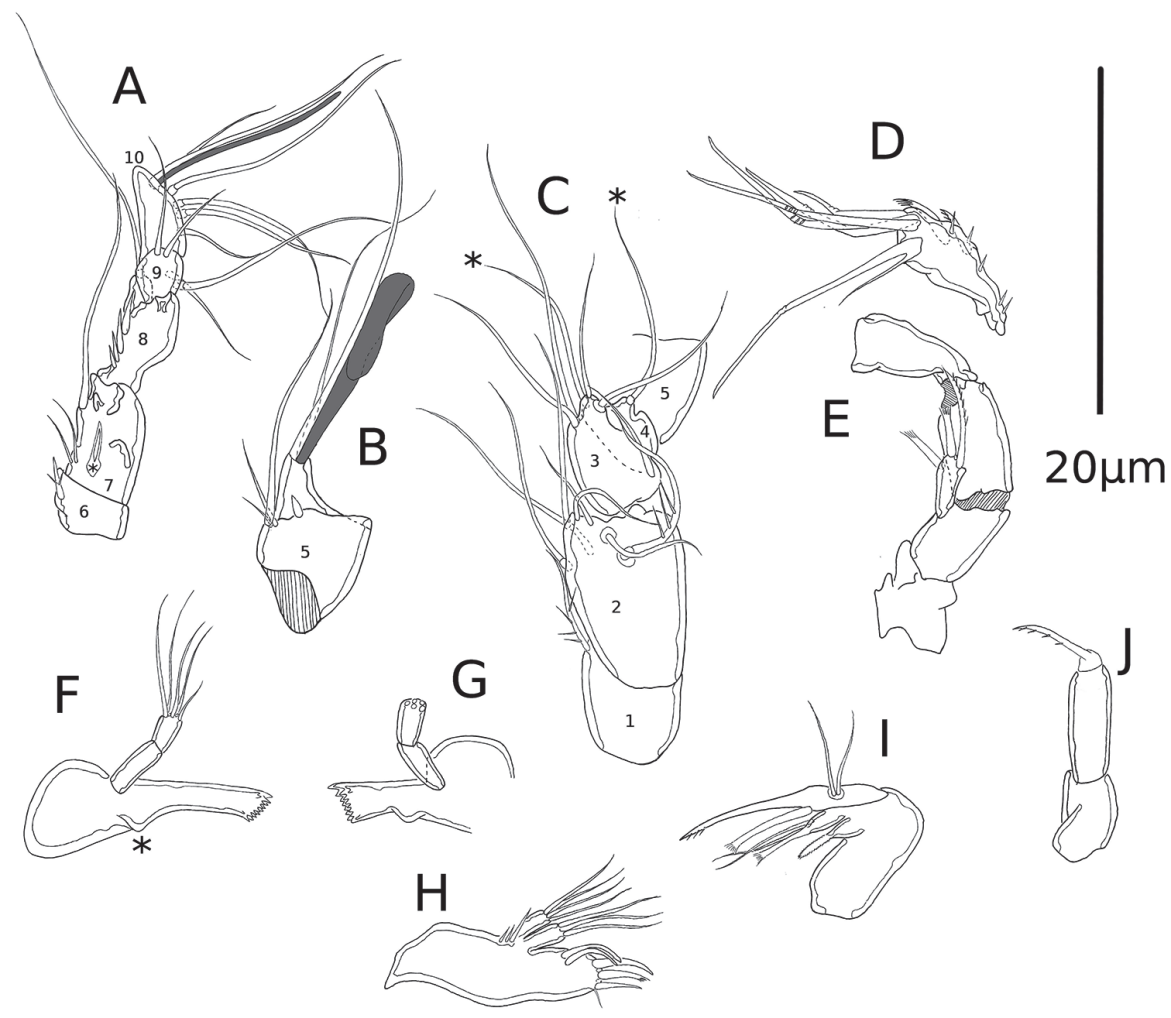
*Psammonitocrella kumeyaayi* sp. nov., male. **A** 6^th^ to 10^th^A1 segments **B** 5^th^A1 segment **C** 1^st^ to 5^th^A1 segments **D**enp2 of A2**E**A2 with unarmed enp2**F**md**G**md with unarmed palp **H**mx1**I**mx2**J**mxp. Asterisk mark the setae of the 4^th^A1 segment, and the md oral bulge.

A1 haplocer (Fig. 2A–C), 10-segmented; armature and ornamentation as follows: 1(1)/2(7)/3(5)/4(2)/5(1ms+4+(1+ae))/6(1ms)/7(3+ms)/8(4ms +1)/9(4)/10(4+acrothek). Acrothek consisting of two setae fused to aesthetasc.

A2 (Fig. 2D, E) basis without abexopodal armature; one-segmented exp with long unipinate seta and two spines with comb tip; enp1 rectangular and smooth; enp2 with two inner marginal spines, four apical geniculated setae, and one geniculate outer seta fused basally to small seta.

Md (Fig. 2 F, G) with oral bulge (marked with asterisk), opposite to md palp. Gnathobase smooth, elongate (Fig. 2F; depicted as shorter in 2G due to a different viewing angle) with weakly developed cutting edge; oral margin without distal seta. Palp uniramous, comprising smooth basis and one-segmented enp with five distal setae.

Mx1 (Fig. 2H). Praecoxa, coxa and basis fused. Praecoxal arthrite rectangular; with row of proximal spinules close to the insertion site of the coxal and basal endites; with three distal spines, a minute oral seta and two surface aboral setae. Coxal endite with three long apical setae. Basis approximately of the same length of coxal endite, with four long apical setae. Enp and exp absent.

Mx2 (Fig. 2I). Syncoxal endite with modified finely bipinnate spine with rounded tip. Basal endite with spine with comb-like tip and fused to endite, and two setae. Enp1 drawn into claw, proximally with accessory spine with comb-like tip; enp2 represented by two setae.

Mxp (Fig. 2J) prehensile, with smooth syncoxa and basis, the former slightly shorter than the latter. Enp represented by long and slightly curved claw, ornamented with medial-distal row of spinules along concave side.

**Figure 3.**
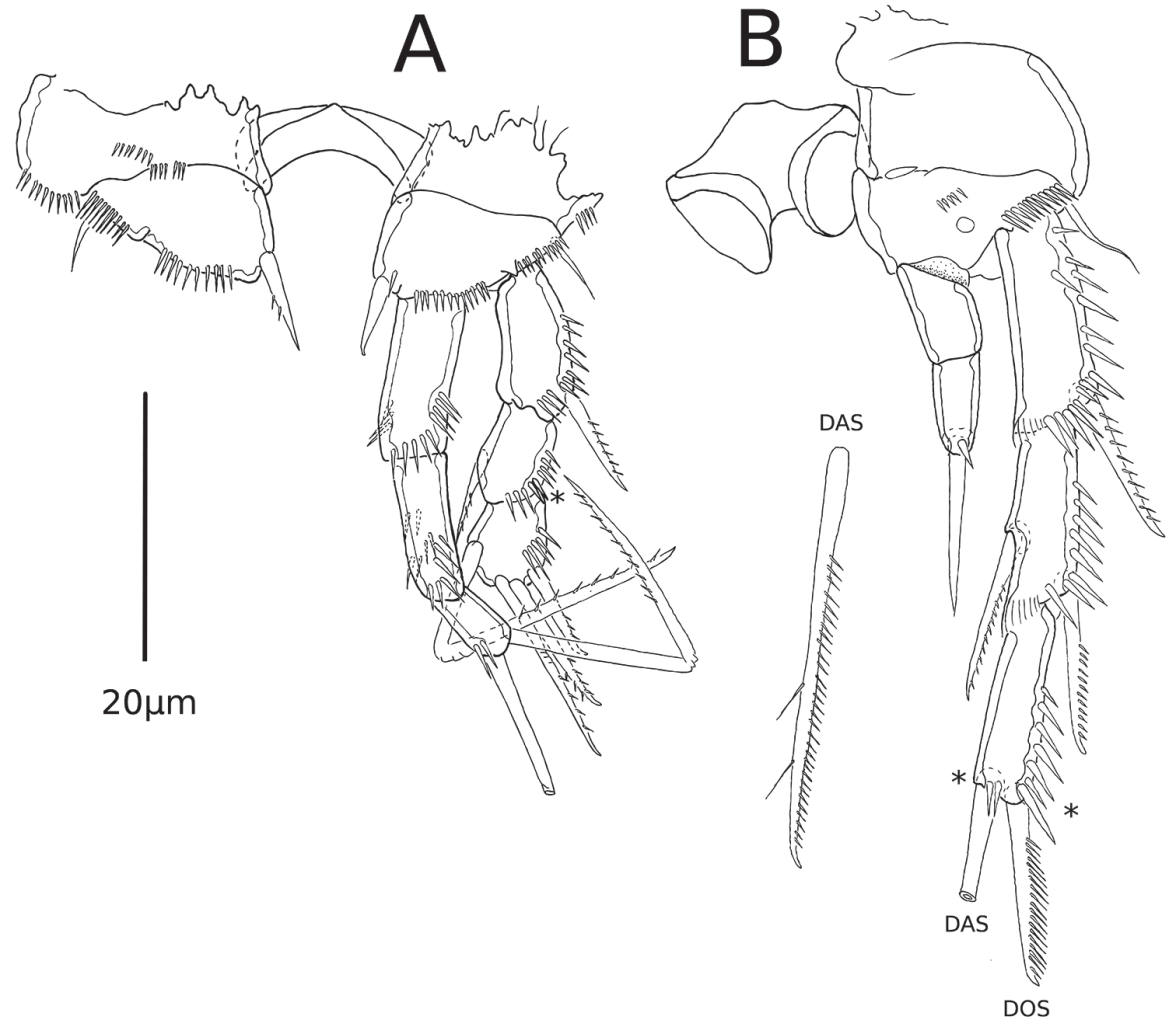
*Psammonitocrella kumeyaayi* sp. nov., male. **A**P1**B**P2. Asterisks mark the position of apomorphies represented by the loss of spines or setae. DAS= Distal apical seta; DOS= distal outer seta.

P1 (Fig. 3A). Intercoxal sclerite smooth, sub-rectangular, wider than long. Coxa with anterior rows of medial, medial-distal, and inner small spinules. Basis with rows of spinules close to insertion site of exp and enp, and at base of outer element; with inner spine and outer seta, the former not sexually dimorphic. Exp three-segmented; exp1 with outer row of spinules and outer unipinnate spine; exp2 without outer spine, with outer row of spinules and with short unipinnate inner seta; exp3 with outer row of spinules, two unipinnate outer spines and two unipinnate and geniculate distal setae. Enp three-segmented; enp1 unarmed, slightly longer than exp1, reaching middle of exp2, with inner, distal and outer rows of spinules; enp2 unarmed, shorter than enp1, with inner, distal, outer and posterior rows of spinules; enp3 shortest, with inner row of spinules, one distal inner and one geniculate distal outer seta, the latter unipinnate distally.

P2 (Fig. 3B). Intercoxal sclerite almost as wide as long, with proximal hump, outer distal extensions rounded, medial-distal part concave. Coxa unornamented. Basis with medial row of spinules, with transverse spinular row close to insertion of exp and at base of outer short seta; with medial pore. Exopodal segments subequal in size; exp1 with outer and distal rows of spinules, with inner distal hyaline frill and unipinnate outer spine; exp2 ornamented as in exp1, with unipinnate outer spine and unipinnate inner spiniform seta; exp3 with outer and distal rows of spinules, with outer unipinnate spine and inner spiniform seta, the latter with pinnate outer margin and with two inner setules. Enp two-segmented, slightly longer than exp1; enp1 unornamented, unarmed; enp2 with small spinule and spiniform distal seta.

**Figure 4.**
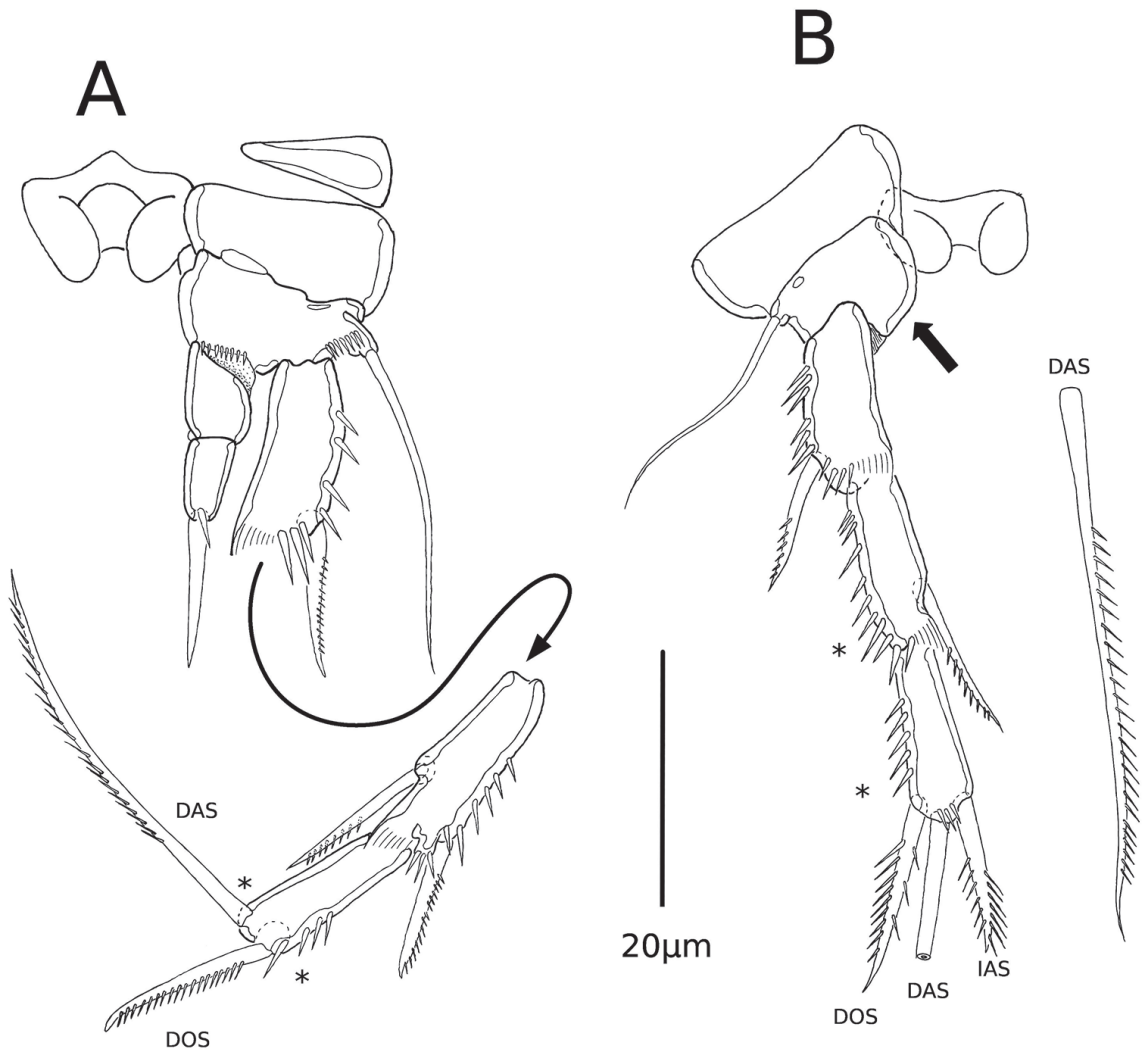
*Psammonitocrella kumeyaayi* sp. nov., male. **A**P3 with detached exp2 and exp3**B**P4. Asterisks mark the position of apomorphies represented by the loss of spines or setae. Arrow indicates the absence of the enp. DAS= Distal apical seta; DOS= distal outer seta; IAS= inner apical seta.

P3 (Fig. 4A) with intercoxal sclerite as in P2. Triangular praecoxa and square coxa unornamented. Basis with row of spinules near insertion of enp, with row of spinules between exp and outer long seta, with proximal outer pore. Exp three-segmented, exp1–3 subequal in size; exp1 with outer and distal rows of spinules, with inner hyaline frill, and outer unipinnate spine; exp2 ornamented as in exp1, with unipinnate outer spine and unipinnate inner spiniform seta; exp3 with outer row of spinules, outer unipinnate spine and inner unipinate seta. Enp two-segmented, almost as long as exp1; enp1 unornamented, unarmed; enp2 with small spinule and spiniform seta distally.

P4 (Fig. 4B). Intercoxal sclerite wider than long, outer distal extensions short, with proximal and distal-medial parts concave. Coxa unornamented. Basis without ornamentation, with proximal outer pore, and outer long seta. Exp three-segmented; exp1–3 subequal in size; exp1 with outer and distal spinules, with inner hyaline frill, and outer unipinnate spine; exp2 ornamented as in exp1, without outer spine, with unipinnate inner spiniform seta; exp3 with outer row of spinules, with outer bipinnate spine, distal long unipinnate seta, and inner bipinnate spine. Enp absent.

P5 (Fig. 1C, D). Rectangular benps united by small intercoxal sclerite, with long outer basal seta, and two small bipinnate spines on endopodal lobe. Exp one-segmented, square, with four elements (setae and spines) of variable shape, size and ornamentation in left (Fig. 1C) and right limbs (Fig. 1D); right exp (Fig. 1C) with long and smooth outer seta, short bipinnate distal spine, and comparatively longer bipinnate distal spine, and inner seta, the latter with tuft of setules distally on both sides; left exp (Fig. 1D) with long and smooth outer seta (shorter than in the right limb), distal seta smooth, a distal tiny element probably representing a reduced spine, and an inner bipinnate seta.

P6 (not shown) represented by unarmed cuticular flap.

**Table 2.** Setal formulae of the swimming legs. Roman numerals represent spines; Arabic numerals represent setae and spiniform setae.

Legs	Basis	Exopod	Endopod
P1	1-I	I-0, 0-1, II-2-0	0-0, 0-0, 0-2-0
P2	1-0	I-0, I-1, 0-I+1-0	0-0, 0-1-0^+^
P3	1-0	I-0, I-1, 0-I+1-0	0-0, 0-1-0^+^
P4	1-0	I-0, 0-1, 0-I+1-I	absent

##### Etymology.

The specific epithet “*kumeyaayi*” refers to the Kumeyaay native American people, who inhabited the area of San Diego County for 10,000 years. Evidence of their presence still remains in San Clemente Canyon.

##### Remarks.

Female unknown. The presence of integumental windows on the third and fourth pedigerous somites was reported by Karanovic and Hancock (2009: 11, table 2; character 2) for most species analyzed by them, including *Eduardonitocrella mexicana*, *S. orghidani*, and their closely related species *Psammonitocrella boultoni* and *P. longifurcata*. This character was, however, never mentioned by Rouch (1992) or illustrated in the original description, and Karanovic and Hancock (2009) did not mention whether they inspected the type material of *Psammonitocrella*. However, it is possible that integumental windows are present on some pedigerous somites within the genus *Psammonitocrella*, as indicated by the presence of lateral integumental windows on the second and third pedigerous somites of *Psammonitocrella kumeyaayi* sp. nov. The probable loss of lateral integumental windows in *Psammonitocrella boultoni* and *P. longifurcata* should be considered as derived. The presence of lateral integumental windows is a potential synapomorphy for a larger group of groundwater ameirids as proposed by Karanovic and Hancock (2009). The absence of the outer spine of the P4exp2 is an autapomorphy for the new species.

## Discussion

According to Karanovic and Hancock (2009), the genus *Psammonitocrella* would have fitted nicely into the original diagnosis of the genus *Stygonitocrella* as defined by Petkovski (1976). However, Petkovski’s (1976) diagnosis is too inaccurate and many other taxa would fit that diagnosis. The careful comparison of the species included within *Stygonitocrella* before Karanovic and Hancock’s (2009) revisionary work, and the species included here in *Psammonitocrella* revealed that both genera differ in the armature of P1exp2, P2–P4exp3, and shape of the furca, with major reductions in the armature of P1–P4 and furca elongation in *Psammonitocrella*. However, one could still assume that *Psammonitocrella* is a junior synonym of *Stygonitocrella*, and that the constituent taxa of the former belong to a derived group within the latter. This assumption is not supported by Karanovic and Hancock (2009). They (Karanovic and Hancock 2009) analyzed the phylogenetic affinities among the species previously attributed to *Stygonitocrella*, and concluded that the generic diagnosis of the genus should be emended and the genus rearranged to include only four species: the type species *S. montana* (Noodt, 1965) from Argentina, *S. dubia* (Chappuis, 1937) and *S. guadalfensis* Rouch, 1985 from Spain, and *S. sequoyahi* Reid, Hunt & Stanley, 2003 from the USA. Other species previously included within *Stygonitocrella* were reallocated into *Eduardonitocrella* Karanovic & Hancock, 2009, *Reidnitocrella* Karanovic & Hancock, 2009, and *Megastygonitocrella* Karanovic & Hancock, 2009. If the hypothesis proposed by Karanovic and Hancock (2009) is accepted, *Psammonitocrella* should be considered as monophyletic and closely related to the monotypic genera *Inermipes* Lee & Huys, 2002, *Neonitocrella* Lee & Huys, 2002, and *Eduardonitocrella* (*Stygonitocrella orghidani* was relegated to *incertae sedis* by Karanovic and Hancock 2009, but appears as the sister group of *Eduardonitocrella* in the same publication). It is our opinion that Karanovic and Hancock (2009) restricted too much the ingroup, including in their analysis only freshwater ameirids with one-segmented P4enp, and one- or two-segmented P2 and P3enp. However, both reductions are known to occur in the Parastenocarididae, and the reduction of the P2–P4enp to a two-segmented structure occurs in or within some interstitial genera of ameirids (i.e., *Leptameira* Huys, 2009; *Parevansula* Guille & Soyer, 1966; *Pseudoleptomesochrella* Lang, 1965; *Psyllocamptus* Scott, 1899) and in some genera assigned to the Leptopontiidae Lang, 1948 (i.e. P2-P4enp, *Bereraia* Huys, 2009; *Leptopontia* Scott, 1902; *Parasewellina* Cottarelli, Saporito & Puccetti, 1986; *Prosewellina* Mielke, 1987; *Psammopsyllus* Nicholls, 1945; *Sewellina* Krishnaswamy, 1956; *Syrticola* Willems & Claeys, 1982).

In fact, the 250+ species of the Parastenocarididae would code in the cladistic analysis of Karanovic and Hancock (2009) with exactly the same character states as in *Psammonitocrella*—Karanovic and Hancock (2009) ignored this taxon—forming a cluster with *Psammonitocrella*, and evidencing their sister-group relationship.

One important achievement of Martínez Arbizu and Moura (1994) was the discovery and justification of the sister-group relationship between the Parastenocarididae and *Psammonitocrella*. In an evolutionary context this is relevant because this evidenced that the Parastenocarididae—one of the largest continental groundwater family of the Harpacticoida—did not evolve from pre-adapted marine interstitial ancestors, but from freshwater interstitial (groundwater) ancestors. This has deep implications for the understanding of the colonization and evolution of groundwater fauna. Because both the Parastenocarididae and Ameiridae had a family rank at that time, placing *Psammonitocrella* as sister group of the Parastenocarididae unequivocally implies its exclusion from the Ameiridae. Martínez Arbizu and Moura (1994) however refrained from giving a full family rank to *Psammonitocrella*, but also from including this genus into the Parastenocarididae, waiting for more robust evidence.

Lee and Huys (2002) listed *Psammonitocrella* as belonging to the Ameiridae without properly discussing Martínez Arbizu and Moura’s (1994) hypothesis or proposing synapomorphies for the inclusion of *Psammonitocrella* into the Ameiridae. However, Lee and Huys (2002) were uncritically followed by subsequent researchers (see Reid et al. 2003; Boxshall and Halsey 2004; Karanovic 2004, 2006; Karanovic and Hancock 2009), and *Psammonitocrella* was considered to belong to the Ameiridae without proper phylogenetic analyses.

The simple removal of a genus from one family to another without proper discussion is naive, and it does not solve the real problem. The phylogenetic position of *Psammonitocrella* cannot be solved without discussing the position of its sister group, Parastenocarididae, nor without including in the discussion the Ameiridae as a whole. Note that also Martínez Arbizu and Moura (1994) did not include all the Ameiridae in their discussion. They just defined the Ameiridae as displaying the synapomorphic modification of the basal inner seta of P1 in the males. It is interesting to note that all Ameiridae discussed by Karanovic and Hancock (2009) display this characteristic sexually dimorphic seta in the males, but this seta is not modified in the Parastenocarididae and *Psammonitocrella*. Martínez Arbizu and Moura (1994) argued consequently for the retention of the plesiomorphic state of this character in the Parastenocarididae and *Psammonitocrella*, rather than a secondary reduction.

The sister-group relationship between the Parastenocarididae and *Psammonitocrella* allows for only two systematic scenarios.

1) Parastenocarididae + *Psammonitocrella* are the sister group of the Ameiridae (or any other family). In this case, a new family should be proposed for *Psammonitocrella*, and the Parastenocarididae would be composed only by those species with the synapomorphic characters proposed by Corgosinho et al. (2007), Ranga-Reddy et al. (2014), and Corgosinho et al. (2017) (e.g., grasping male P3; one-segmented male and female P2-P4enp; absence of outer spine on the P2 and P4exp2 of both male and female, etc.).

or

2) Parastenocarididae + *Psammonitocrella* is a monophyletic group within the Ameiridae. In this case the family rank of the Parastenocarididae would be compromised. “Parastenocarididae” would be a junior subjective synonym of “Ameiridae”, and the Parastenocarididae + *Psammonitocrella* would be a derived group within the Ameiridae.

In an evolutionary context, it would make sense that the Parastenocarididae evolved from a lineage of freshwater ameirids that became interstitial in continental waters and colonized aquifers and groundwaters. The analysis offered by Karanovic and Hancock (2009) convincingly places *Psammonitocrella* as phylogenetically related to a group of freshwater interstitial ameirids, but they unfortunately ignored the Parastenocarididae.

We advocate for the consideration of alternative 2 as the most realistic evolutionary working scenario.

If we accept that *Psammonitocrella* is an Ameiridae, the evidence suggesting its sister-group relationship with the Parastenocarididae—both sharing the loss of the inner seta on the enp1 of the P1—indicates that the Parastenocarididae should be included into the Ameiridae. But the relationships to marine taxa as discussed by Martínez Arbizu and Moura (1994) would remain unresolved. *Psammonitocrella*, Parastenocarididae and Leptopontiidae share the loss of the inner seta of the enp2 of P1 and most importantly, the loss of the outer spine of the exp2 of P1—which is a rare reduction within Harpacticoida (Martínez Arbizu and Moura 1994)—and could indicate that the Leptopontiidae should also be incorporated into the Ameiridae.

According to Boxshall and Jaume (2000), to treat the freshwater ameirids as the product of a single independent colonization event probably represents an underestimate. Therefore, future phylogenetic studies should include both freshwater and marine ameirids, especially those interstitial marine genera with reduced P2–P4enp and reduced inner and outer armature of P2–P4exp3, to test the hypothesis that some freshwater taxa are more related to marine ones, and that the invasion of the fresh water by this family followed multiple waves. In addition, the position of the Parastenocarididae and Leptopontiidae within the ameirid-like harpacticoids should be tested.

The family Parastenocarididae can be easily accommodated within the Ameiridae as the sister group of *Psammonitocrella*. Within the Parastenocarididae the P1enp is reduced to a two-segmented enp [three-segmented P1enp in *Psammonitocrella*], and the inner seta of P1exp2 is lost [present in *Psammonitocrella*]; the enp of P2 and P3 is one-segmented [probably two-segmented in the ground pattern of *Psammonitocrella*], and the exp2 of P2 and P4 lost the inner and outer elements [present in *Psammonitocrella*]. The outer spine of the P4exp2 is absent in the Parastenocarididae and in *P. kumeyaayi* sp. nov. For the mouthparts, the md has a uniramous palp in both *Psammonitocrella* and the Parastenocarididae (two-segmented in the former, without armature on the proximal segment, and one-segmented in the latter), and the mx1 has a reduced distal armature of three spines on the praecoxal arthrite of both taxa. Except for *E. mexicana*, the distal rim of the anal operculum of the species studied by Karanovic and Hancock (2009) reaches the posterior margin of the telson as in the Parastenocarididae.

Taking this into account, future studies are necessary to unfold the relationship between the Ameiridae and Parastenocarididae. We believe that only a phylogenetic analysis of the Ameiridae and Parastenocarididae, with a large taxonomic coverage based on both traditional morphological and multi-gene datasets is suitable to undisputedly determine which one of the two hypotheses mentioned above is better supported by the data. Unfortunately, within the Harpacticoida the taxon sampling for molecular work is still in its infancy.

The monophyly of *Psammonitocrella* can be supported by some reductions of the armature of P1 to P4, and the length of the furca. Of remarkable importance is the cylindrical furca which is also longer than the telson (ap), the basis of P1 in male with unmodified inner spine (ap), loss of the outer spine on the P1exp2 (ap), loss of the outer spine of the exp3 of P2 (ap), P3 (ap) and P4 (ap), loss of the inner apical seta (IAS) on the exp3 of P3 (ap) (Karanovic and Hancock 2009), transformation of the IAS of the P4exp3 into a spine (ap), reduction of the P4enp to a small knob or its complete loss (ap).

Nowadays the genus *Psammonitocrella* is composed of *P. boultoni* and *P. longifurcata* from Sycamore creek (Arizona, USA), and *P. kumeyaayi* sp. nov. from the San Clemente Canyon, San Diego (California, USA). The new species differs remarkably from *P. boultoni*, and *P. longifurcata* in the loss of the outer spine of the P4exp2 (ap), in the presence of a one-segmented P5exp (pl), and in the presence of an outer seta of the basis of P1 (pl) and P2 (pl). The one-segmented exp of the P5 of *P. kumeyaayi* sp. nov. closely resembles that of *P. boultoni*. In the former species it is armed with four elements —one very reduced spine on the left limb—, and three setae are present in the exopodal lobe of the P5 of *P. boultoni*. However, the benp of *P. kumeyaayi* sp. nov. is armed with two spines, but it is armed with only one spine in *P. boultoni*. The enp of the P2 and P3 is two-segmented in both *P. boultoni* and *P. kumeyaayi* sp. nov., whereas the P2 and P3enp is one-segmented in *P. longifurcata*. The shape and length of the furca is similar in *P. boultoni* and *P. kumeyaayi* sp. nov., and it is much longer in *P. longifurcata*.

The two-segmented P2 and P3 enps of *P. boultoni* and *P. kumeyaayi* sp. nov. are definitely plesiomorphic within the genus. The one-segmented P5exp and the presence of two spines on the benp of P5 of *P. kumeyaayi* sp. nov are also considered plesiomorphic. The long furca of *P. longifurcata* seems to be an apomorphic condition within the genus. The presence of the outer seta on the basis of the P1 and P2 are plesiomorphic for *P. kumeyaayi* sp. nov. Considering this, it seems possible that *P. longifurcata* and *P. boultoni* forms a monophyletic group within *Psammonitocrella*, sharing the absence of the outer basal seta of P1 and P2, what could be also supported by the fact that *P. longifurcata* and *P. boultoni* occur in sympatry. The absence of the outer seta on the basis of P1 and P2 is uncommon within Harpacticoida, whilst the reduction or loss of the enp may have occurred convergently many times. Appendage reductions, character losses, and miniaturization (i.e., the evolution of extremely small adults) are common denominators of the ‘darkness syndrome’ of many stygobionts (Galassi 2009). Consequently, the loss of the enp could be a convergent event in both *P. longifurcata* and *P. kumeyaayi* sp. nov. Alternatively, the absence of the P4enp could be the ancestral condition for *Psammonitocrella*, and the small knob representing the P4enp of *P. boultoni* could be attributed to secondary expression, i.e., it is an autapomorphy for this species. However, although reductions such as the loss of an enp could occur convergently within monophyletic groups, without a proper phylogenetic analysis and observation of the types studied by Rouch (1992), it is difficult to establish how the three species of *Psammonitocrella* are related to each other.

### Key to the species of *Psammonitocrella*

**Table:** 

1	Enp2 of P4 with outer spine	**2**
–	Exp2 of P4 without outer spine; enp two-segmented in the P2 and P3; enp of P4 absent; armature of the exp3 of P2–P3 (0-I+1-0); armature of the exp-3 of P4 (0-I+1-I); P5 with an outer seta, benp with two spines, exp one-segmented, with asymmetric armature; telson and furca with long tube pores; furcal ramus about 2.5 longer than wide	***P. kumeyaayi* sp. nov.**
2	Two-segmented enp of P2 and P3; enpP4 reduced to a knob; armature P2 and P3exp3 (0-I+1-0); armature P4exp3 (0-I+1-I); P5 with an outer seta, benp with a single seta, expopodal lobe fused to benp and armed with 3 setae; furcal ramus about 4 times longer than wide	***P. boultoni***
–	One-segmented enp of P2 and P3; enpP4 absent; armature of P2exp3 (0-I+2-0); armature of P3–P4exp3 (0-I+1-0); P5 with an outer seta, reduced into a small plate, with asymmetric armature; furcal ramus almost 7 times longer than wide	***P. longifurcata***

## Conclusions

1) A new species of the freshwater ameirid genus *Psammonitocrella* is proposed for California (USA). The new species can be clearly distinguished from its congeners by the absence of the outer spine on P4exp2 and the presence of a one-segmented P5exp.

2) *P. kumeyaayi* sp. nov. is probably the sister group of a monophylum formed by *P. boultoni* and *P. longifurcata*. However, without the study of the species described by Rouch (1992), it is difficult to establish how the three species are related to each other.

3) If *Psammonitocrella* is an Ameiridae, the evidence suggesting its sister-group relationship with the Parastenocarididae indicates that the Parastenocarididae should be included into the Ameiridae.

4) Future phylogenetic studies should include both freshwater and marine ameirids, to test the hypothesis that some freshwater taxa are more related to marine ones, and that the invasion of the fresh water by this family followed multiple waves. In addition, the position of the Parastenocarididae and Leptopontiidae within the ameirid-like harpacticoids should be tested.

## Supplementary Material

XML Treatment for Psammonitocrella

XML Treatment for Psammonitocrella kumeyaayi
